# Redefining conservative mastectomy: the evolution of surgical techniques

**DOI:** 10.3389/fonc.2025.1575095

**Published:** 2025-03-21

**Authors:** Lorenzo Scardina, Gianluca Franceschini

**Affiliations:** Multidisciplinary Breast Center, Department of Women, Children and Public Health Sciences, Fondazione Policlinico Universitario Agostino Gemelli IRCCS, Rome, Italy

**Keywords:** breast cancer, mastectomy, reconstruction, robotic surgery, surgical technique

The global trend in breast surgery for breast cancer patients is moving toward a de-escalation.

Regarding axillary treatment, the focus has shifted from the possibility of avoiding axillary lymph node dissection (1, 2), to seeing how this approach can also be applied to patients undergoing neoadjuvant chemotherapy (3, 4). Recently, two important prospective trials (5, 6) have shown the possibility of avoiding sentinel lymph node biopsy in selected breast cancer patients.

As for breast surgery, there has been a trend toward performing smaller quadrantectomies, up to procedures where invasive carcinomas no longer require a minimum margin of millimeters, but rather ensuring that resection margins are tumor-free (7). More prospective trials are emerging, suggesting the possibility of avoiding surgery after pCR in breast cancer patients post neoadjuvant chemotherapy (8, 9).

In this global landscape of surgical de-escalation, mastectomy has also undergone significant evolution, though it still remains an important part of clinical practice. Today, unless in specific cases, conservative mastectomy (10) with prosthetic reconstruction is guaranteed, either in a single or two-stage process, or through autologous tissue.

The introduction of nipple-sparing, skin-sparing and skin-reducing mastectomies has significantly improved both the aesthetic outcomes and the psychological well-being of many women (11). However, the benefits of these advanced techniques extend far beyond the preservation of appearance; they present the potential for more precise and less invasive procedures aided by technology, artificial intelligence, and robotic assistance. But what does the future hold for these surgical innovations? And what are the true implications of combining these groundbreaking techniques with cutting-edge technology?

Conservative mastectomies have been considered oncologically safe for at least 15 years now, with preservation of skin and nipple (nipple-sparing or skin-reducing) (12). Prosthetic reconstruction after nipple-sparing mastectomy remains the most frequent, and more specifically, recent studies (13) and reviews (14) have highlighted the superiority of prepectoral implant placement in terms of aesthetic results and quality of life, as well as shorter surgical times and faster postoperative recovery, which translates to economic benefits. Prepectoral reconstruction with polyurethane implants also reduces the need for ADM or mesh use, thereby lowering costs even further.

In the last decade, there has been an increase in the trend of offering bilateral prophylactic mastectomy to patients. This trend can be justified by increased preoperative contrast-enhanced imaging exams, higher genetic risk, onset of tumors at a younger age, and patient choice (15, 16).

It is important to emphasize that while endoscopic and robotic surgery are not yet universally recognized as the standard of care, they can be a viable option in centers of excellence (17).

Robot-assisted mastectomy allows for smaller incisions and greater precision in tissue removal. The robotic system’s ability to translate a surgeon’s hand movements into smaller, more precise movements has led to improved outcomes. Furthermore, robotic system allows better visualization of the surgical area which is crucial in performing nipple-sparing procedures where tissue preservation is key.

Endoscopic technique, which involve using a small camera to guide the surgeon through the procedure, allows for even less invasive approaches to mastectomy.

These minimally invasive surgical techniques provide a magnified view of anatomical structures through 3D visualization, significantly smaller surgical accesses, and faster postoperative recovery times, demonstrating the importance and necessity of utilizing these innovative techniques for highly selected cases (18, 19).

Looking to the future, artificial intelligence (AI) is expected to play a pivotal role in conservative mastectomies, both in preoperative planning and intraoperative navigation. AI-powered imaging systems are already enhancing the surgeon’s ability to detect and map tumors ensuring more accurate gland removal during mastectomy. These systems can analyze mammography, Magnetic Risonance and ultrasound to provide highly detailed 3D reconstructions of the breast allowing for better decision-making regarding surgical technique.

In addition to improving surgical precision, AI has the potential to revolutionize post-operative care. Machine learning algorithms can predict complications based on patient data, such as age, medical history and cancer type providing personalized risk assessments and tailored care plans (20, 21).

In this trend of innovation and advancements in mastectomy techniques, it is essential to remember that the gold standard for early-stage breast cancer, in carefully selected patients, remains breast-conserving surgery combined with radiotherapy (22, 23).

Moreover, thanks to second-level oncoplastic techniques, breast-conserving surgery can now be extended even to cases that would have previously been considered for mastectomy (24).

Several recent studies have also indicated that in patients undergoing neoadjuvant chemotherapy who achieve a pathological complete response (pCR), breast-conserving surgery can be considered a valid alternative to mastectomy (25, 26).

The prospects of conservative mastectomy are undeniably promising. Advances in surgical techniques, reconstructive options, and the integration of robotics, endoscopy, and AI are opening limitless possibilities for improving patient outcomes. Personalized, less invasive treatments will likely become the standard of care offering patients not only the hope of survival but also the confidence and body image they deserve post-surgery. As we continue to push the boundaries of what’s possible, technology will undoubtedly play an increasingly pivotal role in shaping the future of breast cancer treatment.
